# Successful excision of a retrorectal cyst through trans-sacral approach: A case report

**DOI:** 10.1016/j.ijscr.2020.05.023

**Published:** 2020-05-23

**Authors:** Tlal Matouq Alsofyani, Mohammed Yousef Aldossary, Faisal Fahd AlQahtani, Khalid Sabr, Ameera Balhareth

**Affiliations:** a Department of General Surgery, Colorectal Surgery Section, King Fahad Specialist Hospital-Dammam, Saudi Arabia; b Department of General Surgery, King Abdulaziz Hospital, National Guard Hospital, AlAhsa, Saudi Arabia

**Keywords:** Retrorectal cyst, Tailgut cyst, Posterior trans-sacral resection

## Abstract

**Introduction:**

Retrorectal cysts are rare congenital cystic lesions usually diagnosed in middle-aged women. They are generally asymptomatic; however, local pressure may result in complications. Pain or discomfort in the pelvic, sacral, lower back or perianal area are the most common symptoms. The diagnosis is difficult, and multidisciplinary management is required. An epidermoid cyst is the most common type. Surgical resection is the main treatment, and 3 operative approaches are commonly used: abdominal, trans-sacral, and combined abdominosacral. The selection of the approach depends on the nature and location of the lesion. Here, we present a case that demonstrates the trans-sacral approach to a retrorectal cyst is a feasible option in terms of safety and minimal invasiveness for selected patients with this rare type of retrorectal cystic lesion.

**Presentation of case:**

A 38-year-old woman with no comorbidities incidentally showed a retrorectal cyst on magnetic resonance imaging performed during pregnancy. The patient underwent surgical resection under general anesthesia, trans-sacral incision was performed, the posterior rectum exposed, and the cyst removed. No complications were seen in the postoperative period.

**Discussion:**

Posterior trans-sacral resection (Kraske) is preferred for patients with posterior retrorectal cyst because it provides adequate exposure.

**Conclusion:**

Posterior trans-sacral resection allows proximal extension for elimination of the infection and in cases of adherence of the cyst to surrounding structures or in malignancy, which require *en bloc* resection.

## Highlights

• Retrorectal cysts are rare congenital cystic lesions commonly seen in middle-aged women.
• A 38-year-old woman with retrorectal cyst underwent trans-sacral resection.
• She showed no postoperative complications.
• Trans-sacral resection provides adequate exposure of the posterior retrorectal cyst.

## 1 Introduction

Retrorectal cyst (tailgut cyst) is a rare congenital cystic lesion predominantly affecting women [1]. The majority of tailgut cysts are benign [2]. Malignant transformation is reported in 2%–10% of cases [2]. The cysts are usually diagnosed by clinical assessment and magnetic resonance imaging (MRI) [3]. Surgical excision depends on the location of the cyst. For posterior tailgut cyst, trans-sacral resection with an alternative by minimally invasive surgery is the best option [4,5]. The literature on the trans-sacral approach is very limited. Therefore, we present a case demonstrating the trans-sacral approach to a retrorectal tumor as a feasible option in terms of safety and minimal invasiveness selected patients with this rare type of retrorectal cystic lesion. This case report is in line with the SCARE criteria [6].

## 2 Presentation of case

A 38-year-old woman not known to have any medical illness presented to our clinic with a retrorectal cyst that was incidentally found during her pregnancy. She complained of difficulty in voiding. She denied any history of change in bowel habit, hematochezia, and obstructive urinary symptoms. Upon examination, the patient was vitally stable and afebrile. Local examination revealed a palpable bulge at 12 o’clock position extending 5 cm into the coccyx. Abdominal examination, including per rectum examination was unremarkable.

**Fig. 1:**
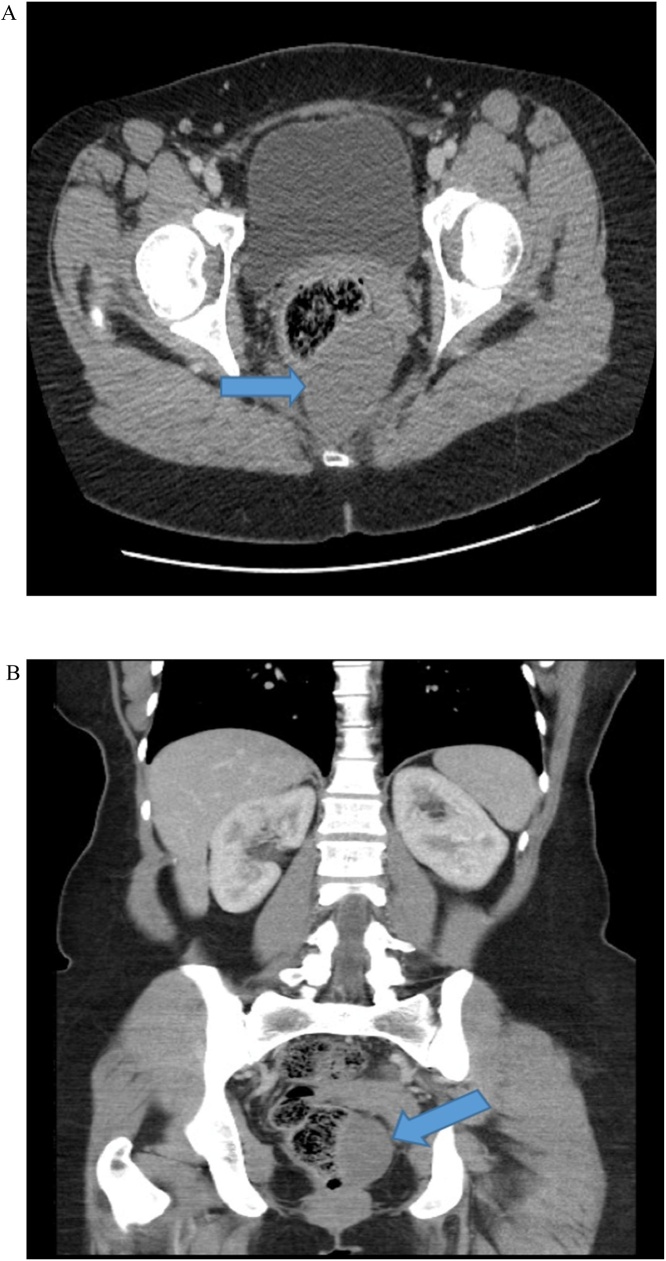
**A:** CT scan of the abdomen revealed a unilocular non-enhancing low-attenuation mass at the retrorectum measuring 5.4 × 4.6 cm. **B:** Coronal view of the same image.

Routine laboratory tests, including complete blood count, hepatic and renal function tests, and coagulation profile, were all within the normal range. Serum tumor markers, including carcinoembryonic antigen and alpha-fetoprotein, were all within the normal range. Computed tomography (CT) of the abdomen revealed a unilocular non-enhancing low-attenuation mass at the retrorectum measuring 5.4 × 4.6 cm (Fig. 1).

**Fig. 2:**
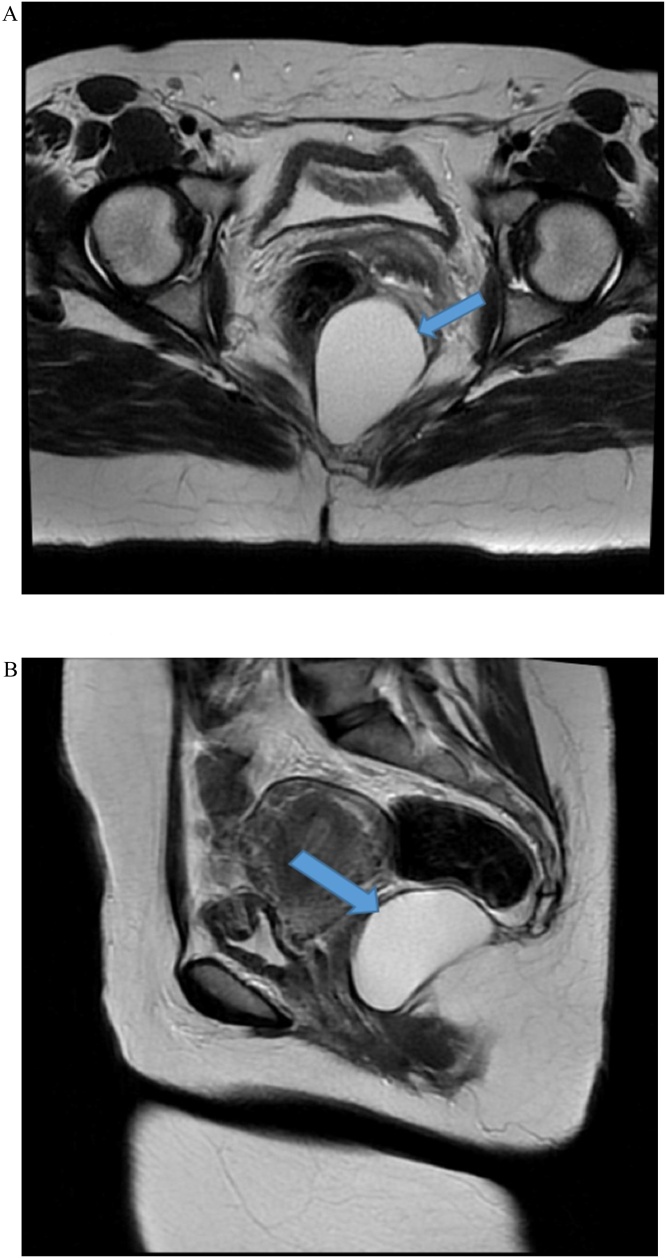
**A:** MRI of abdomen confirmed a well-defined homogeneous at the lower pelvis, retrorectal in location, approaching the level of the anal canal. Anterior displacement of the rectum was seen with no signs of rectal wall signal alteration to suggest infiltration. **B:** Sagittal view of the same image.

MRI confirmed a well-defined homogeneous high T2 intermediate to high T1 signal intensity structure at the lower pelvis, retrorectal in location, approaching the level of the anal canal. Anterior displacement of the rectum was seen with no signs of rectal wall signal alteration to suggest infiltration. Abutting of the inferior margin of the coccyx was seen with no spinal extension or bone destruction (Fig. 2).

**Fig. 3:**
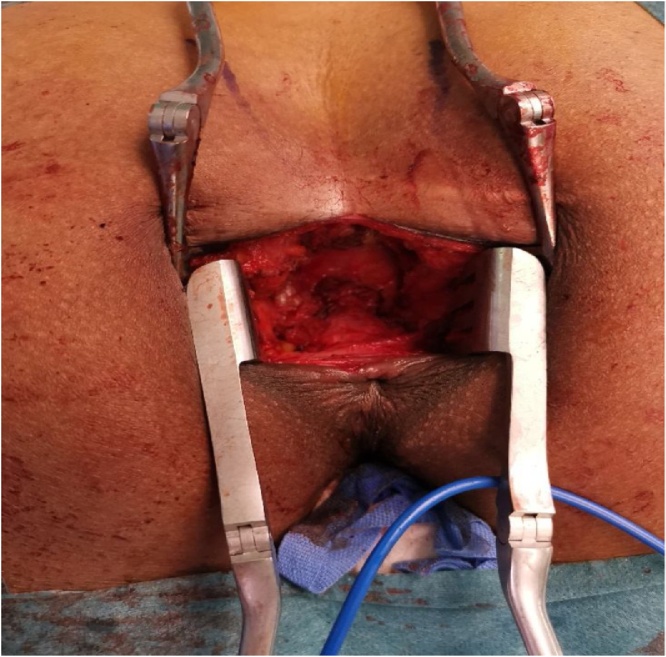
A trans-sacral incision.

**Fig. 4:**
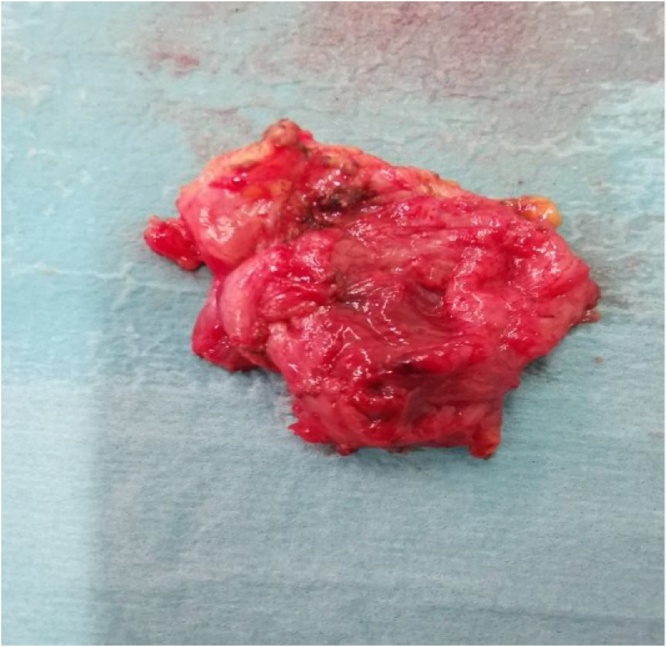
A Retrorectal cyst, measuring 3 × 5 cm, located posterior to the rectum.

**Fig. 5:**
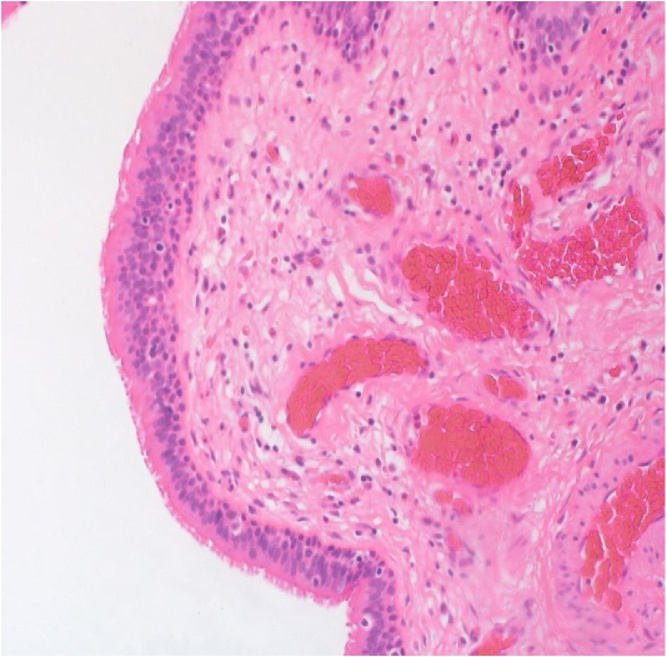
Histological examination revealed a multilocular cystic structure lined by pseudostratified ciliated and transitional epithelium with foci of mucinous differentiation. No teratomatous components were identified. The histopathological features were consistent with tailgut cyst.

Under general anesthesia, the patient was placed in the jackknife position. A trans-sacral incision was made (Fig. 3), and the retrorectal cyst, measuring 3 × 5 cm, located posterior to the rectum (Fig. 4) was completely excised along with a part of her coccyx bone. Thereafter, the wound was closed. Histological examination revealed a multilocular cystic structure lined by pseudostratified ciliated and transitional epithelium with foci of mucinous differentiation. The cyst wall showed smooth and skeletal muscle tissue, adipose tissue, and foci of lymphoid aggregates. An area of histiocytic inflammation was also seen. No teratomatous components were identified, and there was no evidence of malignancy. The histopathological features were consistent with a tailgut cyst (Fig. 5). The postoperative recovery was uneventful, and the patient was discharged home on postoperative day 2 with regular follow-up for 6 months duration in our outpatient department.

## 3 Discussion

Retrorectal cysts, which are rare and usually affect middle-aged women, are typically asymptomatic; however, some patients may present with symptoms caused by the local effects of the cyst, including constipation, rectal fullness, and lower abdominal pain with a retrorectal mass palpable on digital rectal examination [7]. Retrorectal cysts are classified as epidermoid cysts, dermoid cysts, enteric cysts (tailgut cysts and cystic rectal duplication), and neurenteric cysts according to their origin and histopathologic features [7,8]. The cysts are diagnosed by imaging studies, such as CT scan and MRI [9,10]. A well-defined, unilocular or multilocular, thin-walled cystic lesion is the most common imaging feature [10]. CT and MRI used also to visualized the involvement of adjacent organs [11]. Segar et al. describe MRI correctly identified malignancy in 72 of the 76 patients [12].

In mayo clinic experience done over 120 patients showed sigmoidoscopy was useful for large tumors but in the small lesions was negative [13]. In a case series obtained in 10 patients, the transmural mucosal penetration was not detected in any of them [11]. The advantage of flexible sigmoidoscopy is to determine the involvement of the rectal mucosa and to define the proximal extent of the lesion [14]. There is still controversy regarding the preoperative biopsy in retrorectal tumors in the literature. Several approaches were reported in the literature. Trans-peritoneal, trans-retroperitoneal, trans-vaginal, and trans-rectal biopsies should be avoided to minimize the risk of postoperative complications and recurrence [15]. Dozois et al. recommend to performing CT guidance biopsy in the transperineal or parasacral regions because this approach is within the surgical resection field [15]. Biopsy complications including recurrence, infection, fistula, and abscess were found in the mayo clinic experience [13]. Messick et al. reported that there is no increased risk of seeding of tumor cells after the preoperative biopsy [16]. Sung Wook Baek and his colleagues used preoperative anorectal manometry in the patients with fecal incontinence because a total excision of retrorectal cyst will put the patient in high risk of worsening fecal incontinence [17].

Complete surgical resection is the treatment of choice owing to the risk of complications, such as recurrence, local symptoms, and malignancy [18]. Different approaches were used in the literature. Posterior surgical excision through the trans-sacral incision is the preferred treatment for the posterior tailgut cyst [4]. The posterior approach is ideal for retrorectal tumors that do not extend above the level of sacral nerve 3 [[19], [20], [21]]. Posterior trans-sacral approach preferred for small, benign tumors and cases with nerve involvement, given the improved visualization of nerves [20]. Several studies showed the benefits of a minimally invasive approach of such a smaller wound, less post-operative pain, less length of stay, and excellent visualization of pelvic structures [11,21,22]. On the other hand, laparoscopic and robotic resection have been reported in the literature with longer operative time; however, they are not beneficial in cysts suspected to be malignant with increased risk of rupture [4,5,23].

## 4 Conclusion

Trans-sacral excision is a feasible option and safe to perform. It is a minimally invasive and safe option. It allows proximal extension for elimination of the infection and in cases of adherence of the cyst to surrounding structures or in malignancy, which require *en bloc* resection.

## Declaration of Competing Interest

The authors declare no conflict of interest.

## Funding

This study did not receive any funding from governmental or private organizations.

## Ethical approval

Ethical approval was obtained from the Institutional Review Board of the King Fahad Specialist Hospital, Dammam, Saudi Arabia.

## Consent

Written informed consent was obtained from the patient for publication of this case report and accompanying images. A copy of the written consent is available for review by the Editor-in-Chief of this journal on request.

## Author contribution

Study concept or design – AB, TMS, KS.

Data collection – AB, TMS, MYD, FFQ.

Data interpretation – AB, TMS, MYD, FFQ.

Literature review – AB, TMS, MYD, FFQ.

Drafting of the paper – AB, TMS, MYD.

Editing of the paper – AB, TMS, MYD, KS.

## Registration of research studies

Not required.

## Guarantor

Tlal Matouq Alsofyani.

## Provenance and peer review

Not commissioned, externally peer-reviewed.
